# 1,1-Dimethylhydrazine: MAK Value Documentation, addendum – Translation of the German version from 2021

**DOI:** 10.34865/mb5714e9_4ad

**Published:** 2024-12-23

**Authors:** Andrea Hartwig, MAK Commission

**Affiliations:** 1 Institute of Applied Biosciences. Department of Food Chemistry and Toxicology. Karlsruhe Institute of Technology (KIT) Adenauerring 20a, Building 50.41 76131 Karlsruhe Germany; 2 Permanent Senate Commission for the Investigation of Health Hazards of Chemical Compounds in the Work Area. Deutsche Forschungsgemeinschaft, Kennedyallee 40, 53175 Bonn, Germany. Further information: Permanent Senate Commission for the Investigation of Health Hazards of Chemical Compounds in the Work Area | DFG

**Keywords:** 1,1-dimethylhydrazine, carcinogenicity, germ cell mutagenicity, toxicity, DNA methylation

## Abstract

The German Commission for the Investigation of Health Hazards of Chemical Compounds in the Work Area (MAK Commission) has re-evaluated the occupational exposure limit value (maximum concentration at the workplace, MAK value) of 1,1-dimethylhydrazine [57-14-7] considering all toxicological end points. Relevant studies were identified from a literature search. The acute toxicity of 1,1-dimethylhydrazine is caused by depletion of gamma-aminobutyric acid leading to the effects on the central nervous system. After chronic and subchronic exposure, target organs were blood, liver, nervous system, colon and spleen in dogs and liver and colon in rats. In mice, the substance caused lesions in the nose, lungs and gall bladder. In carcinogenicity studies, inhaled 1,1-dimethylhydrazine lead to tumours in the lungs, pituitary gland and pancreas in rats and to thyroid carcinomas, haemangiosarcomas and Kupffer cell sarcomas in mice. Ultra-pure 1,1-dimethylhydrazine induced tumours in the lungs, liver, blood vessels and the nasal mucosa of mice. Orally applied, it caused tumours in the liver and pituitary gland of rats, in the blood vessels, lungs and kidneys of mice and in the blood vessels and colon of hamsters. Therefore 1,1-dimethylhydrazine remains classified in Carcinogen Category 2. No MAK value can be derived. The substance is clastogenic and mutagenic in bacteria and soma cells in vitro and clastogenic in several organs after exposure in vivo. On the basis of these effects and its ability to inhibit DNA synthesis in mouse testes after oral application, 1,1-dimethylhydrazine has been classified in Germ Cell Category 3 A. 1,1-Dimethylhydrazine is irritating to the skin of dogs, guinea pigs and rabbits and to the eyes of rabbits. Due to the low LD_50_ values determined in animals after dermal application and potential genotoxic effects after dermal application, the “H” designation (for substances which can be taken up through the skin in toxicologically relevant amounts) has been retained. Although no studies considering the sensitizing potential are available, the designation with “Sh” (for substances which cause sensitization of the skin) has been retained due to its structural similarity to the known contact allergen hydrazine.

**Table:** 

**MAK value**	**–**
**Peak limitation**	**–**
	
**Absorption through the skin (1973)**	**H**
**Sensitization (1973)**	**Sh**
**Carcinogenicity (1980)**	**Category 2**
**Prenatal toxicity**	**–**
**Germ cell mutagenicity (2020)**	**Category 3 A**
	
**BAT value**	**–**
	
Synonyms	dimazine *N*,*N*-dimethylhydrazine unsymmetrical dimethylhydrazine
Chemical name (IUPAC)	1,1-dimethylhydrazine
CAS number	57-14-7
Structural formula	H_2_N–N(CH_3_)_2_
Molecular formula	C_2_H_8_N_2_
Molar mass	60.10 g/mol
Melting point	–58 °C (IFA 2019)
Boiling point at 1013 hPa	63 °C (IFA 2019)
Density at 20 °C	0.78 g/cm^3^ (IFA 2019)
Vapour pressure at 20 °C	164 hPa (IFA 2019)
log K_OW_	–1.19 (calculated; NCBI 2019)
Solubility	miscible with water (NCBI 2019)
pKa value at 25 °C	7.21 (NCBI 2019)
**1 ml/m^3^ (ppm) ≙ 2.494 mg/m^3^**	**1 mg/m^3^ ≙ 0.401 ml/m^3^ (ppm)**
	
Hydrolytic stability	no data
Production	reaction of monochloramine and dimethylamine (NCBI 2019)
Uses	solvent, intermediate and rocket fuel (NCBI 2019)

Documentation for 1,1-dimethylhydrazine was published in 1973 (Henschler 1973, available in German only). In this addendum, the substance and in particular its carcinogenicity are re-evaluated on the basis of the more extensive data from studies carried out since 1972.

## 1 Toxic Effects and Mode of Action

1,1-Dimethylhydrazine is irritating to the skin of dogs and to the eyes of rabbits.

After inhalation exposure for 6 months, lung adenomas, pituitary adenomas and islet cell adenomas developed in rats at 5 ml/m^3^. In mice, thyroid carcinomas occurred at 0.5 ml/m^3^, haemangiosarcomas and Kupffer cell sarcomas at 5 ml/m^3^. With pure 1,1-dimethylhydrazine, the incidences of lung adenomas, liver adenomas, lymphomas, nasal mucosal adenomas, osteomas and haemangiomas were increased in mice after exposure to 5 ml/m^3^ for 12 months. In rats, pituitary adenomas and hepatocellular adenomas and carcinomas were induced after an oral 1,1-dimethylhydrazine dose of 7.9 mg/kg body weight and day administered for 2 years. In mice, haemangiomas, haemangiosarcomas and renal adenomas were induced at similar doses, and lung tumours developed at and above 2 mg/kg body weight and day. In hamsters, the administration of a 1,1-dimethylhydrazine dose of about 130 mg/kg body weight and day with the drinking water led to angiomas and angiosarcomas, colon tumours and adrenal adenomas; subcutaneous injection of the substance resulted in malignant nerve tumours.

With chronic and subchronic exposure, the target organs are the blood, liver, intestine, nervous system and spleen in dogs, the intestine and liver in rats, and the nose, lungs, gall bladder and endometrium in mice.

Valid studies of developmental toxicity are not available.

A skin sensitizing potential seems likely for 1,1-dimethylhydrazine as hydrazine is a strong contact allergen and cross-reactions between hydrazine derivatives are known (Greim 1999). However, specific studies for 1,1-dimethyl­hydrazine are not available.

Even though the numerous studies that are available are older and were not carried out according to current test guidelines, it is possible to draw the following conclusions with respect to genotoxicity: 1,1-Dimethylhydrazine is mutagenic and clastogenic in bacteria and mammalian cells in vitro. Studies carried out in vivo yielded evidence of DNA-damaging effects including DNA adduct formation in mammalian cells. The results from in vivo studies and consideration of all the data for genotoxicity led to the conclusion that the substance is clastogenic in somatic cells. Furthermore, there is evidence that 1,1-dimethylhydrazine reaches the germ cells and has a clastogenic effect there.

## 2 Mechanism of Action

### 2.1 Neurotoxicity

1,1-Dimethylhydrazine can form hydrazones with derivatives of vitamin B6, which leads to the impairment of processes that require vitamin B6 as a cofactor. A functional change in the gamma-aminobutyric acid receptor was given as an explanation for the central nervous effects that occurred. Accordingly, tremors, vomiting and convulsions occur when lethal or near-lethal doses are administered (ATSDR 1997; Kennedy 2012).

### 2.2 Genotoxic effects and carcinogenicity

During the metabolism of the substance, highly reactive intermediates such as methyl radicals can be formed, which can damage DNA, RNA or other cell components (ATSDR 1997). Thus, methylation of DNA bases with the formation of N7-methylguanine in vivo (Sagelsdorff et al. 1988) and N3-methyladenine, O6-methylguanine and N7-methylguanine in vitro (Kumari et al. 1985) has been demonstrated. DNA-damaging, mutagenic and clastogenic effects occurred in various tests (Section 5.5). These are considered to be causative for the carcinogenic effects of the substance.

### 2.3 Local irritation

Due to its alkaline properties in aqueous solution, 1,1-dimethylhydrazine has a strong irritant effect on the eyes, skin and mucous membranes (ECHA 2019; Kennedy 2012). Concrete evidence for these statements is not available.

## 3 Toxicokinetics and Metabolism

### 3.1 Absorption, distribution, elimination

There are no data from studies in humans.

Monkeys, dogs, cats and rats were given single intraperitoneal injections of ^14^C-1,1-dimethylhydrazine or non-radio­labelled 1,1-dimethylhydrazine. The rats received 10, 20, 30, 40 or 50 mg/kg body weight. In addition, 2 rabbits were given an intravenous dose of 50 mg/kg body weight. Two female cats were treated with 10 mg/kg body weight and 4 female cats with 50 mg/kg body weight. Seven male and 11 female dogs were given 100 mg/kg body weight and monkeys doses of 1, 5, 10, 20, 30, 40 or 100 mg/kg body weight. One animal was used for each of the doses from 1 to 30 mg/kg body weight, and 3 and 15 animals were used for the doses of 40 and 100 mg/kg body weight, respectively. Rapid absorption of the substance into the blood was observed after intraperitoneal injection. Since no radioactivity was detected in the blood plasma of rats 2 to 24 hours after treatment, rapid distribution of the substance in the tissue and rapid elimination can be assumed. Plasma levels in monkeys tended to drop off after 1 hour and were not detectable after 24 hours. In cats and dogs, a similar amount of radioactively labelled substance (30%–50%) was excreted in the urine, consisting of the parent substance. In rabbits, comparatively higher levels of radioactivity were found in the liver and colon (8.9% in the liver, 11.6% in the colon 2 hours after treatment). However, this was not investigated in the other species, as the accumulation of the substance in specific organs or tissues was not suspected (Back et al. 1963).

Two Sprague Dawley rats per group were given single intraperitoneal injections of ^14^C-1,1-dimethylhydrazine at doses of 20, 60 and 80 mg/kg body weight. After 53 hours, 21%, 12% and 19% of the injected radioactivity had been exhaled as ^14^CO_2_, while 56%, 53% and 70% was excreted in the urine and 24%, 35% and 11% remained in the tissues, respectively (Dost et al. 1966).

Six female Sprague Dawley rats were given single intraperitoneal injections of ^14^C-1,1-dimethylhydrazine of 40 mg/kg body weight. After 30 minutes and after 4 hours, the distribution of radioactivity in the tissues and excreta of 3 test animals was examined. Less than 2% of the dose was exhaled. About 19% of the injected radioactivity was recovered in the urine 4 hours after injection. The urine contained the metabolite glucosedimethylhydrazone (3%–10% of the excreted radioactivity) and the parent substance 1,1-dimethylhydrazine (50%–60% of the excreted radioactivity). In addition, 20%–25% of the excreted radioactivity was attributed to an unidentified metabolite. The liver and blood contained 3.7% and 2.7% of the administered radioactivity, respectively, 4 hours after injection. 51.2% remained in the carcass and thus the total recovery was 77.7%. In the animals examined 30 minutes after administration, the radioactivity levels in the urine and carcass were 5.7% and 75.8%, respectively (Mitz et al. 1962).

Rats were given single intraperitoneal injections of ^14^C-1,1-dimethylhydrazine in doses of 20, 40 or 60 mg/kg body weight. Seven hours after treatment, 23%, 19% and 12% of the radioactivity was exhaled as ^14^CO_2_, respectively. In addition, in rats given a single dose of 11 mg/kg body weight, 12% was detected as ^14^CO_2_ 4 hours after administration. At this dose, an additional 25% of the radioactivity was retained in the body and 43% of this was excreted in the urine. Accumulation of the substance in specific organs or tissues was not suspected. The higher percentage of exhaled ^14^CO_2_ compared with the value in the study by Mitz et al. (1962) at the same dose of 40 mg/kg body weight can be explained by a more specific method of determination and a higher number of test animals (NIOSH 1978).

After non-occlusive dermal application (with a glass rod) of 1,1-dimethylhydrazine doses of 0.3, 0.6, 1.2 or 1.8 g/kg body weight to the chest skin of anaesthetized dogs (3–4 crossbreeds per dose), 1,1-dimethylhydrazine was detected in the arterial blood after 30 seconds. The highest concentration was 125 mg/l and was determined 120 to 180 minutes after the application of 1.2 g/kg body weight. The lower doses always resulted in blood levels below 5 mg/l. The maximum concentration in urine was 600 mg/l and was determined after 300 minutes at the dose of 1.2 g/kg body weight. The dose of 1.8 g/kg body weight resulted in only 200 mg/l urine. An attempt was made to prevent additional uptake of the substance by inhalation by placing the animals under a fume hood (Smith and Clark 1971). However, it is unclear whether this actually prevented absorption by inhalation. Therefore, and due to the implausible toxicokinetic data reported, the validity of the study is questionable.

### 3.2 Metabolism

Carbon dioxide (Dost et al. 1966; Mitz et al. 1962) and glucosedimethylhydrazone were detected as metabolites of 1,1-dimethylhydrazine in vivo (Mitz et al. 1962).

In in vitro studies, the formation of formaldehyde and monomethylhydrazine was observed (Prough et al. 1981). Microsomes from rat and hamster liver cells require NADPH and oxygen for the *N*-demethylation of the compound, and the reaction was inhibited by the addition of flavin monooxygenase inhibitors but not by cytochrome P450 inhibitors (Prough et al. 1981). Rat liver slices were incubated with the substance, resulting in degradation to carbon dioxide. Formaldehyde was formed with rat liver microsomes and S9 fraction (Godoy et al. 1983).

In the presence of rat liver microsomes and hepatocytes, methyl radicals were formed from 1,1-dimethylhydrazine, which could be prevented by the inhibition of cytochrome P450 and monooxygenases containing flavin. The authors assume that the methyl radicals originate from the monomethylhydrazine formed (Albano et al. 1993; Tomasi et al. 1987).

In the DNA of liver cells from Sprague Dawley rats, methylation at the N7 position of guanine was detected (Sagelsdorff et al. 1988). From this methylation, an intermediately formed methyldiazonium ion can be inferred as the alkylating species (Augusto et al. 1990).

In in vitro experiments with human fibroblasts, N7-methylguanine and O6-methylguanine as well as N3-methyladenine were detected after exposure to 1,1-dimethylhydrazine (Kumari et al. 1985).

Thus, it appears that flavin monooxygenase-catalysed *N*-demethylation to monomethylhydrazine takes place, from which methyl radicals are formed by flavin monooxygenase or cytochrome P450. Oxidation of the methyl groups produces formaldehyde, which is metabolized to carbon dioxide. In the course of metabolism, the methyldiazonium ion is presumably formed.

**Fig.1:**
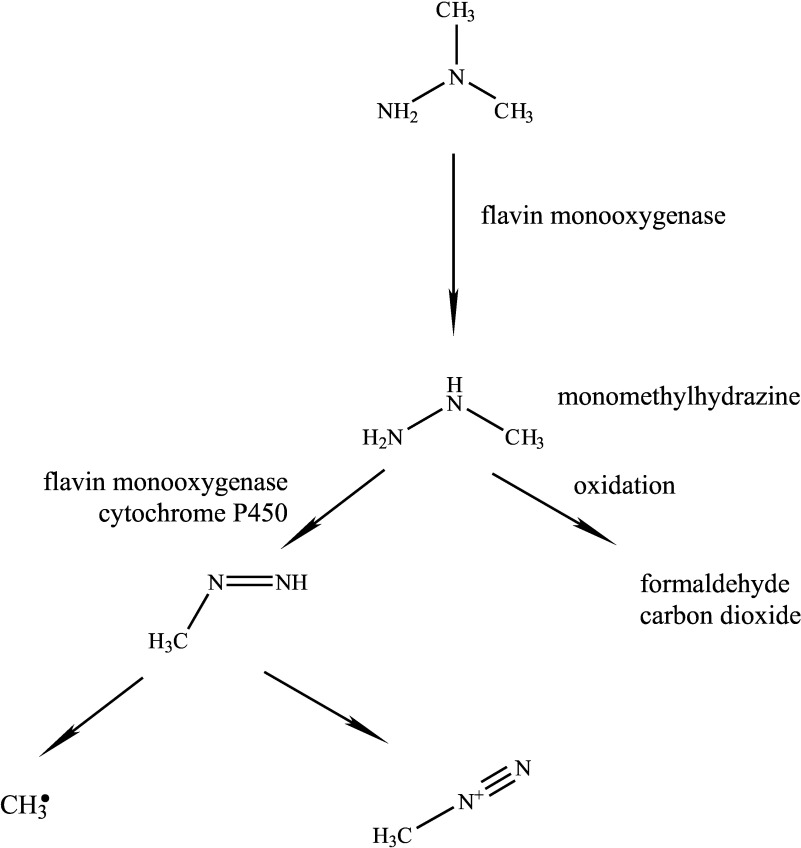
Metabolism of 1,1-dimethylhydrazine

Figure 1 shows the different degradation pathways in the metabolism of 1,1-dimethylhydrazine.

#### 3.2.1 Summary

The rapid uptake of 1,1-dimethylhydrazine into the blood and systemic distribution of the substance in the organism can be assumed. Under the action of cytochrome P450 and flavin-containing monooxygenases, 1,1-dimethylhydrazine may be demethylated and converted into methyl radicals and other highly reactive intermediates (possibly methyldiazonium ions). 1,1-Dimethylhydrazine is exhaled as carbon dioxide and eliminated unchanged or as glucose­dimethylhydrazone in the urine.

## 4 Effects in Humans

### 4.1 Single exposures

A case report describes the medical treatment of a 31-year-old man who was exposed to 1,1-dimethylhydrazine (concentration not reported) in an explosion. In addition to severe burns, the patient suffered respiratory distress and neurological damage manifested by confusion and agitation as well as convulsions. The liver enzyme levels in the blood were also markedly increased. The symptoms were treated successfully by the administration of pyridox. Later, however, signs of polyneuritis developed (Dhennin et al. 1988).

### 4.2 Repeated exposure

A case report describes the exposure of 5 laboratory workers to 1,1-dimethylhydrazine over a period of 6 months at low but not further specified concentrations. In the first 3 months, exposure took place on 6 days per week and 10 hours per day, followed by 6 to 8 additional 4-hour exposures. Hepatotoxicity was determined using the cephalin cholesterol flocculation test (US EPA 2009).

The effects of exposure to an unknown concentration of 1,1-dimethylhydrazine were investigated in 1193 workers in the Danish Air Force. The workers’ blood and urine were examined every 3 or 4 months from 1961 to 1964. Elevated alanine aminotransferase activities were observed in the blood of 47 workers at least at one testing period, indicating damage to the liver caused by the substance (US EPA 2009).

Both studies were already described in the documentation on methylhydrazines from 1973 (Henschler 1973).

Epidemiological carcinogenicity studies with employees at rocket engine test facilities are listed in Section 4.7.

### 4.3 Local effects on skin and mucous membranes

There are no data available.

### 4.4 Allergenic effects

It seems plausible that 1,1-dimethylhydrazine has a skin sensitizing potential as hydrazine is a strong contact allergen and cross-reactions between hydrazine derivatives are known (Greim 1999). However, specific studies with 1,1-dimethylhydrazine are not available.

### 4.5 Reproductive and developmental toxicity

There are no data available.

### 4.6 Genotoxicity

There are no data available.

### 4.7 Carcinogenicity

Exposure to hydrazine increased the incidences of lung cancer and colorectal tumours in the workers of a rocket engine test facility in the United States. The workers (6044 in the cohort observed for mortality with 600 cancer cases, 5049 in the cancer incidence cohort with 691 cancer cases) were exposed to monomethylhydrazine and 1,1-dimethylhydrazine in addition to hydrazine between 1950 and 1993 while handling rocket fuels. A job-exposure matrix was used to assess the exposure of the workers as low (control group, n = 3401 and 2800 workers in the mortality and cancer incidence cohorts respectively), medium (n = 1593 and 1394 workers in the mortality and cancer incidence cohort, respectively) and high (n = 1050 and 850 workers in the mortality and cancer incidence cohort, respectively). If a lag phase of 20 years was taken into account, the relative risk for lung cancer, based on incidence, was 1.18 (95% confidence interval (CI): 0.62–2.24) and 2.49 (95% CI: 1.28–4.86) in the medium and high exposure groups, respectively, compared with the level of risk determined for the low exposure group. The dose–response trend was statistically significant. Smoking status was established only for some of the workers. According to the authors, smoking was not a confounder, because they assumed that there were no differences in smoking status among the examined groups and the risks for the other cancer sites associated with smoking were not increased. In both exposure groups, relative risks of 1.75 (95% CI: 0.93–3.30) and 2.16 (95% CI: 1.02–4.59) were obtained for tumours of the colon and rectum, respectively. The dose–response trend for these tumours was also statistically significant. Even without taking a lag phase into account, the relative risks for these sites were of a similar level and statistically significant in the high exposure group (Ritz et al. 2006).

Another study of this cohort found no increase in mortality for lung cancer (0.89 (95% CI: 0.78–1.02)) or colorectal carcinomas (0.97 (95% CI: 0.75–1.22)) in the entire cohort. The relative risks for 315 test stand mechanics who were assessed as probably having been exposed to hydrazines were 1.67 (95% CI: 0.54–3.89) for colorectal carcinomas and 1.45 (95% CI: 0.81–2.39) for lung cancer (Boice et al. 2006).

The two studies used different methods to evaluate the data of the cohorts to estimate exposure levels. Whereas Ritz et al. (2006) assigned 2643 of 6044 persons to categories with medium or high exposure to hydrazine, Boice et al. (2006), assumed that only 315 of a total of 1642 test stand mechanics had probable exposure to hydrazine based on 8372 test persons who were examined with regard to mortality. Boice et al. (2006) reported exposure mainly to monomethylhydrazine. However, in general it can be assumed, or at least not ruled out, that there was exposure to a mixture of hydrazine, 1,1-dimethylhydrazine and monomethylhydrazine. Therefore, the results support the assumption that the substance causes carcinogenic effects in humans; however, the data obtained are not sufficiently reliable to validate this assumption.

## 5 Animal Experiments and in vitro Studies

### 5.1 Acute toxicity

#### 5.1.1 Inhalation

The acute toxicity of 1,1-dimethylhydrazine was studied in dogs (beagle) after inhalation exposure to concentrations of 24, 52 or 111 ml/m^3^ for 4 hours. The incidence of mortality was 0/3, 1/3 and 3/3 animals in the respective concen­tration groups. Convulsions, vomiting and respiratory distress occurred (ATSDR 1997). For dogs, LC_50_ values of 22 300, 3580 and 981 ml/m^3^ were derived for 5, 15 and 60-minute exposures, respectively. Also convulsions and vomiting were observed in these studies (Weeks et al. 1963).

LC_50_ values of 24 500, 8230, 4010, 1410 and 252 ml/m^3^ were derived for male rats for exposure periods of 5, 15, 30, 60 and 240 minutes, respectively. Clinical signs after exposure ranged from restlessness of the animals to convulsions shortly before death (Weeks et al. 1963).

For mice, an LC_50_ of 172 ml/m^3^ was reported after exposure for 4 hours. Restlessness, dyspnoea, exophthalmos and convulsions were observed (ATSDR 1997).

The LC_50_ for hamsters after 4-hour inhalation exposure was 392 ml/m^3^ (ATSDR 1997).

#### 5.1.2 Oral administration

The LD_50_ value after oral administration was 360 mg/kg body weight for rats (US EPA 2009). Another value for rats of 122 mg/kg body weight was reported (IFA 2019).

#### 5.1.3 Dermal application

Six hours after non-occlusive dermal application (with a glass rod) of 1,1-dimethylhydrazine at doses of 0.3, 0.6, 1.2 or 1.8 g/kg body weight to the chest skin of anaesthetized dogs (3–4 crossbreeds), the incidence of mortality was 1/4, 0/3, 1/3 and 3/3 dogs, respectively. Convulsions and vomiting were observed shortly before death. An attempt was made to prevent additional uptake of the substance via the lungs by placing the animals under a fume hood (Smith and Clark 1971). However, it is unclear whether this actually prevented absorption by inhalation.

LD_50_ values of 770 to 1329 mg/kg body weight were determined after dermal application in rats, rabbits and guinea pigs (IFA 2019).

#### 5.1.4 Summary

The acute toxicity of 1,1-dimethylhydrazine is primarily characterized by effects on the central nervous system in the form of muscle spasms and convulsions, as well as damage to the respiratory tract progressing even to lung collapse, and vomiting and behavioural changes in animal studies (US EPA 2009).

### 5.2 Subacute, subchronic and chronic toxicity

#### 5.2.1 Inhalation

A summary of the experiments with repeated inhalation exposure is shown in Table 1.

Effects on the nerves, blood, liver and spleen and body weight loss were observed in dogs. Respiratory distress and lethargy were seen in rats, convulsions in mice. In addition, hyaline degeneration of the gall bladder and endometrial cysts were detected as non-carcinogenic end points in mice at exposure levels at and above 0.05 ml/m^3^. In the groups of mice exposed to 0.5 and 5 ml/m^3^, damage to the liver (congestion, angiectasis) and lungs (congestion, lymphoid hyper­plasia) was diagnosed. The increase in endometrial cysts observed in mice was concentration-dependent and can be regarded as the most sensitive non-carcinogenic effect of the substance. Damage to the nasal mucosa was observed in mice. Golden hamsters were the least sensitive species. No substance-related effects could be found in this group of animals (Haun et al. 1984). Since rodents and dogs were not examined histopathologically until 18 months and 5 years after the end of exposure, respectively, it is not possible to derive a NOAEC (no observed adverse effect concentration).

**Tab.1:** Effects of 1,1-dimethylhydrazine after repeated inhalation exposure

Species, strain, number per group	Exposure	Findings	References
dog, beagle, 3 ♂	**13 weeks** 0, 25 ml/m^3^, **26 weeks** 0, 5 ml/m^3^, 6 hours/day, 5 days/week, purity not specified	**5 ml/m^3^:** body weight loss, RBC, Hb, Hct ↓, indirect bilirubin ↑, haemosiderosis in spleen; **25 ml/m^3^:** body weight loss, mortality 1/3, convulsions, salivation, diarrhoea, vomiting, fever, weakness, breathing difficulties, movement coordination disorders, Hb, Hct ↓, haemosiderosis in spleen and liver	Rinehart et al. 1960
rat, Wistar, 20 ♂	**6 weeks** 0, 140 ml/m^3^, **7 weeks**, 0, 75 ml/m^3^, 6 hours/day, 5 days/week, purity not specified	**75 ml/m^3^:** shortness of breath, lethargy; **140 ml/m^3^:** tremor, body weight gains ↓, mortality 1/20	
mouse, CF-1, 30 ♀	**6 weeks** 0, 140 ml/m^3^, **7 weeks** 0, 75 ml/m^3^, 6 hours/day, 5 days/week, purity not specified	**75 ml/m^3^:** convulsions, mortality 8/20; **140 ml/m^3^:** mortality 29/30, body weight gains ↓, tremor	
dog, beagle, 4 ♂, 4 ♀, 16 ♀ controls	**6 months** 0, 0.05, 0.5, 5 ml/m^3^ (with 0.12% DMNA), 6 hours/day, 5 days/week, 5 years observation, purity not specified	**5 ml/m^3^:** during exposure: ALT ↑, BSP retention ↑	Haun et al. 1984
hamster, Syrian golden hamster, 200 ♂, 400 controls	**6 months** 0, 0.05, 0.5, 5 ml/m^3^ (with 0.12% DMNA), 6 hours/day, 5 days/week, 18 months observation, purity not specified	**up to 5 ml/m^3^:** no effects caused by 1,1-dimethylhydrazine	
rat, F344, 200 ♂	**6 months** 0, 0.05, 0.5, 5 ml/m^3^ (with 0.12% DMNA), 6 hours/day, 5 days/week, 18 months observation, purity not specified	**0.05 ml/m^3^:** liver: fat deposits	
mouse, C57BL/6, 400 ♀, 800 ♀ controls	**6 months** 0, 0.05, 0.5, 5 ml/m^3^ (with 0.12% DMNA), 6 hours/day, 5 days/week, 18 months observation, purity not specified	**0.05 ml/m^3^ and above:** hyaline degeneration of gall bladder, endometrial cysts; **0.5 ml/m^3^ and above:** lung damage; **5 ml/m^3^** liver: angiectasis	
mouse, C57BL/6, 200 ♀	**12 months** 0, 5 ml/m^3^ (with < 35 µg DMNA/l), 6 hours/day, 5 days/week, 12 months observation, purity not specified	**5 ml/m^3^:** body weights ↓, nose: inflammation, hyperplasia, dysplasia, squamous metaplasia; anus: erosion, prolapse; damage to vascular system, hyaline degeneration of the gall bladder (not statistically significant), cysts of the endometrium and ovaries (not statistically significant)	

ALT: alanine aminotransferase; BSP: bromosulphthalein; DMNA: dimethylnitrosamine; Hb: haemoglobin; Hct: haematocrit; RBC: red blood cells

#### 5.2.2 Oral administration

In an unpublished study, F344 rats (70 animals per sex and group) were exposed to 1,1-dimethylhydrazine concentrations of 0, 1, 50 or 100 mg/l drinking water for 24 months. The average daily doses were 0, 0.07, 3.2 or 6.2 mg/kg body weight for the male rats and 0, 0.1, 4.5 or 7.9 mg/kg body weight for the female rats. Body weights were reduced in a statistically significant manner in the female rats of the middle and high dose groups. Reduced drinking water intake was observed in males of the middle and high dose groups. Mortality was 36%, 36%, 28% and 18% in the males and 32%, 24%, 28% and 10% in the females, respectively. Haematological examinations did not reveal treatment-related findings. In the females, cloudy corneas were observed at and above 4.5 mg/kg body weight and day. A NOAEL (no observed adverse effect level) of 0.1 mg/kg body weight and day and a LOAEL (lowest observed adverse effect level) of 4.5 mg/kg body weight and day for female rats were reported (US EPA 2009). The study was not available to the Commission or the US EPA in the original.

CD-1 mice (90 animals per sex and group) received 1,1-dimethylhydrazine concentrations of 0, 40 or 80 mg/l drinking water for 24 months. The average daily doses were 0, 7.34 or 13.71 mg/kg body weight in the male mice and 0, 11.59 or 21.77 mg/kg body weight in the female mice. The incidence of mortality was 70%, 76% and 98% of the animals in the male mice and 58%, 92% and 92% in the female mice. Drinking water consumption was reduced and food intake sporadically decreased in exposed animals of both sexes. The body weights of the females were reduced in a statistically significant manner (10%) in the high dose group compared with those in the control group. Statistically significant changes in haematological parameters were observed in the males, which were not further specified. These changes started after 6 months in the high dose group and after 12 months in the low dose group. Alanine aminotransferase and sorbitol dehydrogenase levels in the blood increased in the exposed female and male animals. Gross pathological findings included increased liver lobulation in treated males and development of lung and liver nodules in treated males, beginning at 8 months, and in treated females, beginning at 12 months. Chronic multifocal inflammation (at 12–24 months), hypertrophy and necrosis were diagnosed in treated males. In both treated groups of both genders, hemosiderosis and splenic haematopoiesis occurred (US EPA 2009). A NOAEL was not obtained. The study was likewise not available to the US EPA in the original.

In another unpublished study, CD-1 mice (90 animals per group and sex) were exposed to 1,1-dimethylhydrazine concentrations of 0, 1, 5 or 10/20 (♂/♀) mg/l drinking water for 24 months. The average daily intake was 0, 0.19, 0.97 or 1.9 mg/kg body weight for male mice and 0, 0.27, 1.4 or 2.7 mg/kg body weight for female mice. Ten mice per sex and group were examined after 6, 12, 18 and 24 months. A statistically significant increase in mortality occurred only in the high dose group of males (60%, 50%, 64% and 76%, respectively). No dose-related body weight changes were found. An increased incidence of lung nodules was observed in the males in the middle dose group (47%) compared with an incidence of only 21% in the animals of the control group and of 26% in the high dose group (US EPA 2009). The study was not available to the US EPA in the original.

#### 5.2.3 Dermal application

There are no data available.

#### 5.2.4 Intraperitoneal administration

Intraperitoneal injection of 1,1-dimethylhydrazine in a dose of 10 mg/kg body weight and day, 5 days a week, for 4 weeks resulted in an increase in the glucose level in blood plasma and fat deposition in the liver in rhesus monkeys (Patrick and Back 1964).

In another study, groups of 10 female Sprague Dawley rats were given intraperitoneal injections of 1,1-dimethyl­hydrazine at doses of 0, 10, 30, 50 or 70 mg/kg body weight and day for 21 days. Mortality occurred at and above 30 mg/kg body weight and day. Haematological examinations did not reveal any unusual findings. Fat deposition in the tubular epithelium of the kidney occurred in the surviving animals of the 50 and 70 mg/kg groups. These effects were less pronounced in the group given 50 mg/kg body weight and day than at 70 mg/kg body weight and day. Increased aspartate aminotransferase activities in the blood were observed in all treated groups (Cornish and Hartung 1969).

#### 5.2.5 Summary

The target organs of repeated dose administration are the nervous system, liver, blood and lungs. In mice, endometrial cysts and hyaline degeneration of the gall bladder were observed at comparatively low concentrations of 1,1-dimethylhydrazine (0.05 ml/m^3^ and above); nasal mucosal damage was observed at 5 ml/m^3^. At higher concentrations, mortality was increased. Golden hamsters can be considered the least sensitive species and mice the species most susceptible to damage from 1,1-dimethylhydrazine. A NOAEC cannot be given.

### 5.3 Local effects on skin and mucous membranes

#### 5.3.1 Skin

In an acute toxicity study in rabbits and guinea pigs, 1,1-dimethylhydrazine was applied once occlusively to the dorsal skin for 24 hours. No damage to the skin was observed (Rothberg and Cope 1956). After non-occlusive application of 1,1-dimethylhydrazine doses of 0.3, 0.6, 1.2 or 1.8 g/kg body weight to the chest skin of dogs (crossbreeds), distinct reddening of the skin occurred, which faded 10 to 15 minutes after the application of the substance (Smith and Clark 1971).

REACH registrants classified 1,1-dimethylhydrazine as corrosive to the skin (Skin Corr. 1B (H314)) due to the high pH (ECHA 2019), although no such effect was observed in the available studies and the pKa value does not indicate high alkalinity.

#### 5.3.2 Eyes

In a study with rabbits, 3 µl of 1,1-dimethylhydrazine was applied to the left eye of each animal (10 animals in total). Conjunctivitis and erythema of the eyelids were observed 48 hours after application; the effects disappeared within 5 days (no other details; Rothberg and Cope 1956).

### 5.4 Allergenic effects

There are no data available.

### 5.5 Reproductive and developmental toxicity

#### 5.5.1 Fertility

There are no generation studies available.

Groups of 4 to 6 BC3F1/CUM mice (C57BL♀×C3Hf♂, 11–18 weeks old) were given intraperitoneal injections of 1,1-dimethylhydrazine in aqueous solution (doses of 12.5, 31.25, 50, 69, 87.5 mg 1,1-dimethylhydrazine/kg body weight and day, equivalent to 10%, 25%, 40%, 55% or 70% of the LD_50_ of 125 mg/kg body weight) on 5 days. Sperm morphology and the sperm count were examined 0.8 and 3 weeks after the end of exposure in half of the animals, respectively. A dose-dependent increase in the number of abnormally shaped sperm and a reduction in the sperm count after exposure were observed compared with the control values. An additional 50 male BC3F1/CUM mice were given intraperitoneal injections of 1,1-dimethylhydrazine at the dose of 12.5 mg/kg body weight and day for 5 days. Five mice were killed each week and examined. An increased number of abnormally shaped sperm (amorphous, subnormal, twin-tailed and folded) was observed, which returned to the values of the control group after week 4 (Wyrobeck and London 1973).

In a study, 11 to 14-week-old male ((C57BL/6×C3H/He) F1) mice were given 1,1-dimethylhydrazine in aqueous solution by intraperitoneal injection for 5 consecutive days. The doses were in the range from 10 to 100 mg/kg body weight and day. Four dose groups of 4 mice each were studied. The mice were killed for the purpose of sperm morphology examin­ations after 1, 4 and 10 weeks. No abnormal deformations of the spermatozoa were observed (Wyrobek and Bruce 1975).

In another study, possible changes in sperm morphology were investigated in ((C57BL/6×C3H/He) F1) mice given intraperitoneal injections of 1,1-dimethylhydrazine at doses of 0, 62.5, 125, 250, 375 or 500 mg/kg body weight (presumably cumulative doses) in aqueous solution for 5 consecutive days. On day 35 after the final dose, the mice were killed and examined. No damage to spermatozoa was observed (Bruce and Heddle 1979).

#### 5.5.2 Developmental toxicity

The possible developmental toxicity of 1,1-dimethylhydrazine was investigated in F344 rats. In this study, groups of 14 to 18 pregnant rats were given intraperitoneal injections of 1,1-dimethylhydrazine in saline at doses of 0, 10, 30 or 60 mg/kg body weight and day from gestation days 6 to 15. On gestation day 20, the animals were killed and the following parameters were examined: the number and position of implantations, the number of dead and live foetuses, and the number of resorptions. Malformations of the foetuses (external, visceral and skeletal) were additionally examined. Lower weight gains were observed in the dams, compared with those in the vehicle control group; the effect was dose-dependent. In the high dose group, foetal weights were reduced, the number of resorptions was increased and visceral and skeletal malformations were found. According to this study, 1,1-dimethylhydrazine is not teratogenic but embryotoxic (Keller et al. 1984). Due to the intraperitoneal administration route, a direct effect of the substance on the foetus cannot be excluded. Therefore, the study is not included in the evaluation.

#### 5.5.3 Summary

There are no studies of the developmental and reproductive toxicity of 1,1-dimethylhydrazine after inhalation exposure. After intraperitoneal injection, the substance was found to be embryotoxic. However, due to the intraperitoneal administration route, a direct effect of the substance on the foetus cannot be excluded. Therefore, the study is not included in the evaluation.

### 5.6 Genotoxicity

#### 5.6.1 In vitro

##### 5.6.1.1 Bacteria and yeasts

Indicator tests for differential killing provided evidence of genotoxic effects of 1,1-dimethylhydrazine in DNA repair-deficient mutants of Bacillus subtilis (Suter and Jaeger 1982) and Escherichia coli (De Flora et al. 1984; Poso et al. 1995; Suter and Jaeger 1982) without the addition of a metabolic activation system.

As pointed out in OECD Test Guideline 471 (OECD 2020), the Salmonella typhimurium strains TA97, TA98, TA100, TA1535, TA1537 and TA3538 do not provide reliable evidence of mutagenic effects that are caused by hydrazines. Therefore, the use of the Salmonella typhimurium strain TA102 and Escherichia coli WP2 is recommended for the detection of mutagenic effects induced by this group of substances. The mutagenicity tests available for 1,1-dimethylhydrazine confirmed these findings. With a few exceptions, the studies with the Salmonella strains TA100, TA1535, TA1537 and TA1538 yielded negative results. In one study in each case, 1,1-dimethylhydrazine was found to have a mutagenic effect with the strains G46, TA2638 and TA97. Two positive results and one negative result were obtained with the strain TA1530. The mutagenicity of 1,1-dimethylhydrazine could be demonstrated with the strains TA102 and TA98. Mutagenicity was not found in a study in Escherichia coli WP2 (uvrA^-^) (Brusick and Matheson 1976), while 2 tests with and without plasmid pKM101 yielded positive results (Hemminki et al. 1980; Watanabe et al. 1996). 1,1-Dimethylhydrazine likewise yielded positive results in Escherichia coli ada mutants following activation by chemical oxidation (Sedgwick 1992).

The mutagenicity of 1,1-dimethylhydrazine was shown in the ascomycete Aspergillus nidulans with activation in a growth mediated assay, while the result of the plate incorporation test was negative (Bignami et al. 1981). No mutagenic effect was induced in yeast cells (Saccharomyces cerevisiae, D4) (Brusick and Matheson 1976).

##### 5.6.1.2 Mammalian cells

###### Indicator tests

In studies in human dermal fibroblasts, no DNA single-strand breaks were detected with 1,1-dimethylhydrazine. However, binding of methyl groups to bases of the DNA in the form of N7-methylguanine and O6-methylguanine as well as N3-methyladenine was detected, which was, however, of a very low degree. Less than 1% of the radioactiv­ity used was detected as methyl adducts, most of the ^14^C was incorporated into newly synthesized DNA (Kumari et al. 1985). 1,1-Dimethylhydrazine caused DNA single-strand breaks in rat hepatocytes (Sina et al. 1983). However, the val­idity of the study is questionable, as the assessment of cytotoxicity was not conclusive. With an alternative method for the detection of DNA damage and repair (nick translation), induction was observed in human dermal fibroblasts at 300 µM. Simultaneous incubation with the DNA repair inhibitor cytarabine (ara-C) had no effect on the induced damage or its repair (Snyder and Matheson 1985). In human WI38 lung cells, an assay for the induction of DNA repair synthesis (UDS) with metabolic activation yielded positive results. The induced UDS decreased at the highest concentration used, possibly due to concurrent cytotoxicity (Brusick and Matheson 1976). Further UDS tests in cultured primary hepatocytes from ACI/N rats and C3H/HeN mice yielded a negative (rat) and a positive result (mouse) after exposure to 1,1-dimethylhydrazine (Mori et al. 1988).

###### Clastogenicity and mutagenicity tests

A chromosomal aberration test demonstrated a clastogenic effect of 1,1-dimethylhydrazine in hamster ovary cells (IARC 1999).

In a TK^+/–^ mutation assay with L5178Y mouse lymphoma cells without metabolic activation using thymidine excess as a selection agent, 1,1-dimethylhydrazine induced small but not large colonies, indicating a clastogenic effect. Testing for ouabain, thioguanine or cytosine arabinoside resistance mutation yielded negative results (Rogers and Back 1981). In another TK^+/–^ gene mutation assay with L5178Y mouse lymphoma cells, a clear, dose-dependent increase in resistant cell colonies with the selection agent bromodeoxyuridine was found with and without the addition of a metabolic activation system. Here, however, no distinction was made between large and small colonies (Brusick and Matheson 1976).

The induction of gene mutations at the *hprt* locus of V79 cells was evident after treatment of the cells with rat liver perfusate or bile when 1,1-dimethylhydrazine had previously been added to the perfusion fluid. Without this activation, 1,1-dimethylhydrazine was not mutagenic (Beije et al. 1984). The data from in vitro tests are shown in detail in Table 2.

**Tab.2:** In vitro studies of the genotoxicity of 1,1-dimethylhydrazine

End point	Test system	Concentration [µg/plate]^a)^	Effective concentration [µg/plate]^a)^	Cytotoxicity [µg/plate]^a)^	Results		References
					–m. a.	+m. a.	
indicator tests differential killing bacteria	Bacillus subtilis H17/M45, Bacillus subtilis HLL3g/HJ-15 (spot test and agar incorporation test)	serial dilutions up to the limit of solubility, no other details	no data		+	n. t.	Suter and Jaeger 1982
	Escherichia coli AB1157, Escherichia coli JC5547, Escherichia coli JC2921, Escherichia coli JC2926 (spot test and agar incorporation test)		no data		+	n. t.	
	Escherichia coli AB1157, Escherichia coli JC5519 (only agar incorporation test)		–		–	n. t.	
	Escherichia coli WP2 trpE56, Escherichia coli CM871 trpE65 uvrA155 recA56 lexA (modified spot test)	WP2: 1202–12 020 CM871: 60.1–601	no data		+	n. t.	Poso et al. 1995
gene mutation bacteria Escherichia coli	Escherichia coli WP2, Escherichia coli WP67 uvrA^– ^polA^–^, Escherichia coli CM871 uvrA^– ^recA^– ^lexA^– ^(liquid micromethod)	4–8 serial dilutions up to the limit of solubility, no other details	no data	toxicity limit taken into account for the concentrations, serial dilutions up to the limit of solubility, no other details	+	+	De Flora et al. 1984
	Escherichia coli WP2 uvrA^–^ (spot test)	0, 5.0 µl/plate	–	no data	–	–	Brusick and Matheson 1976
	Escherichia coli WP2 uvrA (pre-incubation test)	20–10 000 µM	no data	+	+	n. t.	Hemminki et al. 1980
	Escherichia coli ada mutants, chemical oxidation as activation mechanism	2 mM	2 mM	+	–	+	Sedgwick 1992
	Escherichia coli WP2/pKM101, Escherichia coli WP2uvrA/pKM101 (plate incorporation test)	0, 63, 125, 250, 313, 500, 625, 1000, 1250, 2500, 5000	≥ 1000	+	+	n. t.	Watanabe et al. 1996
gene mutation Salmonella typhimurium	Salmonella typhimurium TA98 (plate incorporation test and spot test)	0, 0.01, 0.1, 1, 5 µl/plate	–	5 µl/plate	–	–	Brusick and Matheson 1976
	Salmonella typhimurium TA100 (plate incorporation test and spot test)		–	5 µl/plate	–	–	
	Salmonella typhimurium TA1535 (plate incorporation test and spot test)		–	5 µl/plate	–	–	
	Salmonella typhimurium TA1537 (plate incorporation test and spot test)		–	5 µl/plate	–	–	
	Salmonella typhimurium TA1538 (plate incorporation test and spot test)		–	5 µl/plate	–	–	
	Salmonella typhimurium TA98 (plate incorporation test)	0, 0.05, 0.5, 5, 50, 500	500	no data	–	+	Bruce and Heddle 1979
	Salmonella typhimurium TA100 (plate incorporation test)		no data	no data	–	+	
	Salmonella typhimurium TA1535 (plate incorporation test)		–	no data	–	–	
	Salmonella typhimurium TA1537 (plate incorporation test)		–	no data	–	–	
	Salmonella typhimurium TA98 (pre-incubation test)	< 250 µmol/plate	–	no data	–	–	Bartsch et al. 1980
	Salmonella typhimurium TA100 (pre-incubation test)		–	no data	–	–	
	Salmonella typhimurium TA1530 (pre-incubation test)		no data	no data	+	+	
	Salmonella typhimurium TA1535 (pre-incubation test)		no data	no data	+	+	
	Salmonella typhimurium TA1538 (pre-incubation test)		–	no data	–	–	
	Salmonella typhimurium G46 (pre-incubation test)		no data	no data	+	+	
	Salmonella typhimurium TA98 (plate incorporation test)	up to solubility limit/toxicity limit	≥ 1202		+	+	De Flora 1981; De Flora et al. 1984
	Salmonella typhimurium TA100 (plate incorporation test)		–		–	–	
	Salmonella typhimurium TA1535 (plate incorporation test)		–		–	–	
	Salmonella typhimurium TA1537 (plate incorporation test)		–		–	–	
	Salmonella typhimurium TA1538 (plate incorporation test)		–		–	–	
	Salmonella typhimurium TA98 (plate incorporation test)	0, 21, 42, 83, 167, 333 µmol/plate	≥ 42 µmol/plate	167 µmol/plate (–S9) 333 µmol/plate (+S9)	+	+	Parodi et al. 1981
	Salmonella typhimurium TA100 (plate incorporation test)		–	no data	–	–	
	Salmonella typhimurium TA1535 (plate incorporation test)		–	no data	–	–	
	Salmonella typhimurium TA1537 (plate incorporation test)		–	no data	–	–	
	Salmonella typhimurium TA1538 (plate incorporation test)		–	no data	–	–	
	Salmonella typhimurium TA100 (pre-incubation test)	0–451 growth period before treatment 11 hours	no data	no data	+	–	Matsushita et al. 1993
	Salmonella typhimurium TA102 (pre-incubation test)	0–601 growth period before treatment 5, 7 or 11 hours	no data maximum number of revertants after 5-hour growth period	no data	+	–	
	Salmonella typhimurium TA97 (plate incorporation test)	0, 1.62, 8.0, 40, 200, 1000, 5000	no data	no data	+	–	Nielsen et al. 1992
	Salmonella typhimurium TA98 (plate incorporation test)		no data	no data	+	–	
	Salmonella typhimurium TA100 (plate incorporation test)		40	at ≥ 1000	+	+	
	Salmonella typhimurium TA102 (plate incorporation test)		200	5000	+	+	
	Salmonella typhimurium TA1530 (plate incorporation test)		–	no data	–	–	
	Salmonella typhimurium TA1535 (plate incorporation test)		–	no data	–	–	
	Salmonella typhimurium TA1537 (plate incorporation test)		–	no data	–	–	
	Salmonella typhimurium TA102 (pre-incubation test)	0, 75, 150, 300	300	no data	+	n. t.	Poso et al. 1995
	Salmonella typhimurium TA1535 (pre-incubation test)	0, 100, 200, 500, 1000 µg/plate	100	1000	+	(+)	Rogan et al. 1982
	Salmonella typhimurium TA1537 (pre-incubation test)		100	1000	+	+	
	Salmonella typhimurium TA1530 (plate incorporation test)	0, 10, 20, 50 mg/plate	20 mg/plate	50 mg/plate	(+)	n. t.	Tosk et al. 1979
	Salmonella typhimurium TA102 (plate incorporation test)	0, 313, 625, 1250, 2500, 5000	625	no data	+	n. t.	Watanabe et al. 1996
	Salmonella typhimurium TA2638 (plate incorporation test)	0, 78, 156, 313, 625, 1250, 2500, 5000	≥ 625	no data	+	n. t.	
gene mutation fungi	Aspergillus nidulans 35, induction of methionine suppressors and 8-azaguanine resistance (plate incorporation test), growth-mediated metabolic activation	0, 0.1, 0.25, 0.5 µl/plate	0.1 µl/plate	no data	–	+	Bignami et al. 1981
	Saccharomyces cerevisiae D4 (spot and plate incorporation test)	0, 0.01, 0.1, 1, 5 µl/plate, spot test: 5 µl/plate	–	5 µl/plate	–	–	Brusick and Matheson 1976
indicator tests mammalian cells	DNA single-strand breaks (alkali-labile lesions), human foreskin fibroblasts	up to 6.83 mM	–	ED_50_ 6.83 mM	–	n. t.	Kumari et al. 1985
	DNA single strand breaks, alkaline elution, rat hepatocytes	0, 0.03–3 mM	0.03 mM	determination of cytotoxicity not valid	+	n. t.	Sina et al. 1983
	covalent DNA binding, human foreskin fibroblasts	0.5 mM			(+)^b)^	n. t.	Kumari et al. 1985
	DNA repair, nick translation assay, human fibroblasts	0, 0.3 mM	0.3 mM	+ (DNA damage) after 2 hours	+	n. t.	Snyder and Matheson 1985
	UDS, rat hepatocytes (ACI)	0, 0.01–1 mM	–	not cytotoxic in the tested range	–	n. t.	Mori et al. 1988
	UDS, mouse hepatocytes (C3H/HeN)	0, 0.01–1 mM	≥ 0.1 mM	not cytotoxic in the tested range	+	n. t.	
	UDS, human embryonic lung cells (WI-38)	0, 0.1, 0.5, 1.0 µg/ml	≥ 0.1 µl/ml	> 1.0 µg/ml	–	+	Brusick and Matheson 1976
chromosomal aberrations mammalian cells	hamster ovary cells (CHO)	0, 20 µg/ml	20 µg/ml	no data	+	+	IARC 1999
gene mutation mammalian cells	TK^+/–^ test, mouse lymphoma cells (L5178Y), selection agent: bromodeoxyuridine	0, 0.01, 0.05, 0.1, 0.25 µl/ml (–m. a.) 0, 0.005, 0.01, 0.05, 0.1 µl/ml (+m. a.)	–m. a.: 0.1 µl/ml; +m. a.: 0.01 µl/ml	+	+	+	Brusick and Matheson 1976
	TK^+/–^ test, mouse lymphoma cells (L5178Y), selection agent: thymidine excess	0, 0.1, 1, 2.5, 5 mM	0.1 mM	at 1 mM about 30% toxicity	+ (small colonies)	n. t.	Rogers and Back 1981
	mutation to ouabain, thioguanine and cytosine arabinoside resistance, mouse lymphoma cells (L5178Y)	0, 0.1, 1, 2.5, 5 mM	–	no data	–	n. t.	
	*hprt* test hamster lung fibroblasts (V79) m. a.: liver perfusate or bile (selenium-deficient and selenium-supplemented rats)	0, 5 mM	5 mM		–	+^c)^	Beije et al. 1984

a) unless otherwise stated

b) < 1% of the radioactivity as methylated bases N7-methylguanine, O6-methylguanine, N3-methyladenine

c) positive with liver perfusate from selenium-deficient and selenium-supplemented rats and with bile from selenium-supplemented rats

–: negative result; +: positive result; (+): only a small positive effect; m. a.: metabolic activation; UDS: DNA repair synthesis test

#### 5.6.2 In vivo

The data from the in vivo genotoxicity tests are shown in Table 3.

##### 5.6.2.1 Drosophila

In the somatic eye mosaic test, larvae of Drosophila melanogaster were examined for possible interchromosomal mitotic recombination. The exposure concentrations were 2.5 and 5 mM 1,1-dimethylhydrazine in physiological saline. Mutagenicity was demonstrated. The high concentration resulted in reduced survival (Vogel and Nivard 1993).

In the SLRL (sex-linked recessive lethal mutations) test – a germ cell test with Drosophila melanogaster – the substance was fed to the flies with and without the addition of inhibitors of various enzymes of metabolism (1-phenylimidazole, iproniazid, *N,N*-dimethylbenzylamine). 1,1-Dimethyhydrazine was not mutagenic. The simultaneous treatment or pre-treatment with 1-phenylimidazole and the pre-treatment with phenobarbital led to a weak increase in the incidence of mutations (Zijlstra and Vogel 1988).

##### 5.6.2.2 Bacteria in vivo–ex vivo

In a host-mediated assay using NMRI mice and the Salmonella typhimurium strain TA1950, 1,1-dimethylhydrazine was not mutagenic (von Wright and Tikkanen 1980).

##### 5.6.2.3 Mammals

###### Indicator tests

Alkaline elution was used to investigate the induction of DNA strand breaks in the liver and lungs of mice after single intraperitoneal injections of 1,1-dimethylhydrazine of 250 mg/kg body weight or after injections on 5 days of 42 mg/kg body weight and day. In the liver, no DNA strand breaks were induced. Single doses did not increase DNA damage in the lungs; after administration for 5 days, the result was positive (Parodi et al. 1981).

For a comet assay, male CD1 mice were treated either intraperitoneally with 50 mg/kg body weight or by gavage with 50 or 100 mg/kg body weight. Three hours after intraperitoneal injection, the induction of DNA strand breaks in the liver and lungs was observed. After oral administration, DNA damage was observed in the liver, stomach and colon as well as in the lungs (only in the high dose group) at and above 50 mg/kg body weight; 24 hours after treatment, the results were negative (Sasaki et al. 1998).

Sprague Dawley rats were given gavage doses of 15 or 23 mg [^14^C]-labelled 1,1-dimethylhydrazine/kg body weight or 0.2, 2 or 20 mg [^3^H]-labelled 1,1-dimethylhydrazine/kg body weight. Dose-dependent binding of methyl groups to DNA bases in liver cells was observed. About 3% to 4% of the radioactivity in the DNA bases was detected in the form of N7-methylguanine; the vast majority is thus incorporated into the DNA bases via biosynthesis. Dimethylnitrosamine was 100 to 700-fold more effective (Sagelsdorff et al. 1988).

The result of a UDS test (autoradiography) in kidney cells of male F344 rats was negative after a single intraperitoneal injection of 50 mg 1,1-dimethylhydrazine/kg body weight. The authors mention that this is a cytotoxic dose (Tyson and Mirsalis 1985).

In a study, described only as a summary, with rats given 1,1-dimethylhydrazine as a single gavage dose of 200 mg/kg body weight, the UDS test in hepatocytes yielded a negative result (no other data; Beije and Olsson 1990).

##### 5.6.2.4 Clastogenicity tests

###### Somatic cells

No clastogenic effects of 1,1-dimethylhydrazine could be detected in an assay for nuclear aberrations (micronuclei, pyknotic nuclei, fragmented nuclei) using colon cells from male and female mice (C57BL/6J). The animals were given gavage doses of 0, 25, 50 or 100 mg/kg body weight (Wargovich et al. 1983).

In a chromosomal aberration test, groups of 5 male rats (“breedless”) were exposed to concentrations of 0, 205, 410 or 1028 mg/m^3^ either once or on 10 consecutive days for 1 hour. Five rats were given gavage doses of 0, 5.4, 10.8 or 26.8 mg/kg body weight either once or 10 times. The analysis of the chromosomal aberrations in bone marrow was performed 24 hours after the (last) treatment. A clastogenic effect of 1,1-dimethylhydrazine was found. This was more pronounced after oral than after inhalation exposure and also more pronounced after repeated administration (Carlsen et al. 2009). The study is considered to have only limited validity because the description of the method is insufficient, the rat strain used is not defined and the results were not presented as individual data. In addition, cytotoxicity was not determined simultaneously. Furthermore, it is unclear whether gaps were taken into account in the evaluation.

A micronucleus test with bone marrow cells from male mice cannot be used for the evaluation due to the lack of a negative control (Suzuki et al. 1994).

In another micronucleus test, a clastogenic effect was observed in the spleen of male mice given intraperitoneal doses of 1,1-dimethylhydrazine of 13.8 to 55 mg/kg body weight. The evaluation 14 days after the treatment revealed stronger induction of micronucleated splenocytes compared with that found in the evaluation 2 days after treatment. Even the lowest dose (13.8 mg/body weight) led to a doubling of micronucleated cells; however, the number did not increase dose-dependently at higher doses (Benning et al. 1994).

Female mice ((C57BL/6×C3H/He) F1) were given intraperitoneal 1,1-dimethylhydrazine doses of 0, 62.5, 125, 250, 375 or 500 mg/kg body weight (presumably cumulative doses) on 5 consecutive days. Four hours after the last dose, the animals were killed. No clastogenic effects were observed in the bone marrow (Bruce and Heddle 1979). The authors reported that about 1000 reticulocytes were analysed per dose group. According to OECD Test Guideline 474 (OECD 2016 a), about 4000 reticulocytes should be counted per animal.

In a micronucleus test in CD1/CR mice, 1,1-dimethylhydrazine doses of 14, 28 or 56 mg/kg body weight were administered twice at intervals of 24 hours via intraperitoneal injection and the animals were then subjected to partial hepat­ectomy. The incidence of micronucleated hepatocytes was increased even at 14 mg/kg body weight. (Cliet et al. 1989).

In a micronucleus test in CD1 mice treated once intraperitoneally with 1,1-dimethylhydrazine doses of 0, 28, 56 or 83 mg/kg body weight, no clastogenic effects were observed in bone marrow cells (Cliet et al. 1993).

###### Germ cells

The studies investigating sperm anomalies (Bruce and Heddle 1979; Wyrobeck and London 1973; Wyrobek and Bruce 1975) are described in more detail in Section 5.5.1. In general, these studies should be evaluated critically, as changes in sperm morphology are not reliable indicators of actual mutations and the relevance of the effects with regard to germ cell mutagenicity is questionable (ICPEMC 1983; Salamone 1988; Wild 1984). The positive results can thus be interpreted only as a cytotoxic effect and cannot be used to assess possible germ cell mutagenicity.

In a micronucleus test with spermatids of mice (CD1), a clastogenic effect after intraperitoneal injections of 0, 28, 56 or 83 mg 1,1-dimethylhydrazine/kg body weight was demonstrated after administration of the doses 6 times (Cliet et al. 1993).

In a dominant lethal test in ICR mice given intraperitoneal injections of 1,1-dimethylhydrazine (0, 1.25, 4.2, 12.5 mg/kg body weight and day) on 5 consecutive days, no clastogenic or aneugenic effects were observed (Brusick and Matheson 1976). The study deviated from OECD Test Guideline 478 (OECD 2016 b) in so far as the number of animals was too low and the mating period (8 weeks instead of 10 weeks) too short. In addition, the high dose was equivalent to 1/10 of the LD_50_ due to the toxicity of 1,1-dimethylhydrazine. This might have been too low to detect a clastogenic or aneugenic effect. For these reasons, the dominant lethal test has not been included in the evaluation of germ cell mutagenicity.

Another dominant lethal test in ICR/Ha mice with higher single intraperitoneal 1,1-dimethylhydrazine doses (25 or 63 mg/kg body weight) likewise yielded negative results (Epstein et al. 1972). At that time, this test was not carried out as specified in the OECD test guideline that was published later (OECD 2016 b); too few animals were used and the results were not reported in detail.

**Tab.3:** In vivo studies of the genotoxicity of 1,1-dimethylhydrazine

Test system		Dose	Results	Remarks	References
**Drosophila melanogaster**					
SMART somatic mutations and reciprocal recombinations	Eye Mosaic Test, Drosophila melanogaster (*white/white*^*+*^*)*	0, 2.5, 5 mM	+	5 mM: decrease in survival	Vogel and Nivard 1993
SLRL sex-linked recessive lethal mutations	Drosophila melanogaster	0, 6, 7.5 mM, in the feed or 20 mM injected	– (without inhibitor)	feed: weakly significant increase after co-/pre-treatment with 4 mM 1-phenylimidazole (enzyme inhibitor) or 12 mM phenobarbital (after pre-treatment)	Zijlstra and Vogel 1988
**In vivo–ex vivo**					
host-mediated assay	mouse (NMRI), groups of 4 ♂, (Salmonella typhimurium TA1950)	0, 140 mg/kg body weight, gavage	–		von Wright and Tikkanen 1980
**Mammals**					
indicator tests	DNA strand breaks, mouse (Albino Swiss), 6 ♂ per group, lungs, liver, alkaline elution	0, 250 mg/kg body weight, intraperitoneal, once	–		Parodi et al. 1981
	DNA strand breaks, mouse (Albino Swiss), 6 ♂ per group, lungs, liver, alkaline elution	0, 42 mg/kg body weight and day, intraperitoneal, 5 days	+	lungs: + liver: –	
	DNA damage, comet assay, mouse (CD1), groups of 4 ♂, liver, lungs, kidneys, brain, bone marrow, bladder, large intestine, gastric mucosa, tissue sampling after 3 and 24 hours	0, 50 mg/kg body weight, intraperitoneal, once	+	liver and lungs: + after 3 hours other organs: –	Sasaki et al. 1998
	DNA damage, comet assay, mouse (CD1), groups of 4 ♂, examined tissues see above	0, 50, 100 mg/kg body weight, gavage, once	+	50 mg/kg body weight and above: liver, stomach and large intestine + after 3 hours 100 mg/kg body weight: lungs + after 3 hours other organs: –	
	covalent DNA binding, rat (SD), 1 ♂ per dose, liver, tissue sampling after 24 hours	0, 0.2, 2, 20 mg [methyl-^3^H]-1,1-DMH/kg body weight; 15, 23 mg [^14^C]-1,1-DMH/kg body weight, gavage, once	+	N7-methylguanine, dose-dependent (about 3%–4% of the radioactivity in the DNA bases)	Sagelsdorff et al. 1988
	UDS test, rat (F344), 3 ♂ per dose, kidneys, tissue sampling after 2 und 12 hours	0, 50 mg/kg body weight, intraperitoneal, once	–	cytotoxic (no other details)	Tyson and Mirsalis 1985
	UDS test, rat, liver, no other details	0, 200 mg/kg body weight, gavage, once	–	selenium supplementation in the diet: no effect on UDS induction	Beije and Olsson 1990
**Somatic cells**					
nuclear aberrations (micronuclei, pyknotic nuclei, fragmented nuclei)	mouse (C57BL/6J), 3 ♂, 3 ♀ per dose group, colon, tissue sampling after 24 hours	0, 25, 50, 100 mg/kg body weight, gavage, once	–		Wargovich et al. 1983
chromosomal aberrations	rat (“breedless”, no other details), 5 ♂ per concentration, bone marrow	0, 205, 410, 1028 mg/m^3^, inhalation, 1 hour/day once	+	increased at 205 mg/m^3^ and above, at 1028 mg/m^3^ statistically significant, questionable validity (see text)	Carlsen et al. 2009
	rat (“breedless”, no other details), 5 ♂ per concentration, bone marrow	0, 205, 410, 1028 mg/m^3^, inhalation, 1 hour/day 10 days	+	at 205 mg/m^3^ and above, questionable validity (see text)	
	rat (“breedless”, no other details), 5 ♂ per dose group, bone marrow	0, 5.4, 10.8, 26.8 mg/kg body weight, gavage, once	+	at 5.4 mg/kg body weight and above, questionable validity (see text)	
	rat („breedless“, no other details), 5 ♂ per dose group, bone marrow	0, 5.4, 10.8, 26.8 mg/kg body weight and day, gavage, 10 days	+	at 5.4 mg/kg body weight and above, questionable validity (see text)	
micronucleus test	mouse (CD1/CR), 5 ♂ per dose group, spleen, tissue sampling after 2 and 14 days	0, 13.8, 27.7, 55.5 mg/kg body weight, intraperitoneal, once	+	at 13.8 mg/kg body weight and above, 14 days after treatment stronger induction compared with 2 days after, 1000 binuclear cells counted, cytotoxicity not investigated	Benning et al. 1994
	mouse (BALB/c AnNCrj), 5 ♂ per dose group, bone marrow, tissue sampling 54 hours after administration of PGE2	20 mg/kg body weight, intraperitoneal, once, ± 3, 10, 30 mg PGE2/kg body weight	–	negative without pre-treatment or with 3, 10 mg PGE2/kg body weight, positive with 30 mg PGE2/kg body weight, not valid because no negative control included	Suzuki et al. 1994
	mouse (CD1/CR), 5 ♂ per dose group, bone marrow, tissue sampling 24 hours after last dose	0, 28, 56, 83 mg/kg body weight and day, intraperitoneal, 1× daily, 2 days	–	PCE/NCE not changed, no cytotoxicity	Cliet et al. 1993
	mouse (CD1/CR), 5 ♂ per dose group, liver	0, 14, 28, 56 mg/kg body weight and day, intraperitoneal, 1× daily, 2 days, 24 hours thereafter partial hepatectomy, 96 hours thereafter evaluation	+	positive at 14 mg/kg body weight and above, strongest effect at 28 mg/kg body weight (56 mg/kg body weight = 50% of LD_50_), cytotoxicity not determined	Cliet et al. 1989
	mouse (C57BL/6×C3H/He), 4 ♀ per dose group, bone marrow	0, 62.5, 125, 250, 375, 500 mg/kg body weight (presumably cumulative), intraperitoneal, 1× daily, 5 days	–	only about 1000 reticulocytes analysed per dose group, no determination of cytotoxicity	Bruce and Heddle 1979
**Germ cells**					
micronucleus test	mouse (CD1/CR), 5 ♂ per dose group, spermatids	0, 28, 56, 83 mg/kg body weight, intraperitoneal, 2× daily, 3 days	+	increase in incidence of micronuclei at 83 mg/kg body weight statistically significant, no cytotoxicity (testis weight)	Cliet et al. 1993
dominant lethal test	mouse (ICR/Ha) 7–9 ♂ per dose group, each male animal mated with 3 females for 8 consecutive weeks, each week 3 other females used	0, 25, 63 mg/kg body weight, intraperitoneal, once	–	LD_50_ 126 mg/kg body weight, at 63 mg/kg body weight 3 animals died; questionable validity (see text)	Epstein et al. 1972
	mouse (ICR), 10 ♂ per dose group, each male animal mated with 2 females on 5 days for 8 consecutive weeks, 3 other females used every week	0, 1.25, 4.2, 12.5 mg/kg body weight and day, intraperitoneal, 5 days	–	LD_50_ 125 mg/kg body weight, questionable validity (see text)	Brusick and Matheson 1976

–: negative result; +: positive result; DMH: dimethylhydrazine; NCE: normochromatic erythrocytes; PCE: polychromatic erythrocytes; PGE2: prostaglandin E2; UDS: DNA repair synthesis

#### 5.6.3 Accessibility of the germ cells

In micronucleus tests with spermatids of mice (CD1), a clastogenic effect in the spermatids was demonstrated after intraperitoneal injection (Cliet et al. 1993). However, this administration route does not allow any conclusion to be drawn on the accessibility of the germ cells via systemic distribution.

However, the possible inhibition of DNA synthesis in the testes of male mice after exposure to 1,1-dimethylhydrazine was investigated. For this purpose, 3 to 4 male mice received a single oral dose of the substance of 200 mg/kg body weight. Subsequently, the inhibition of DNA synthesis was determined according to the Friedman dust method using ^3^H-thymidine. The incorporation of thymidine into the DNA of the mouse testis was reduced to 40.8% of the control value and thus the accessibility of the germ cells after oral administration has been demonstrated (Seiler 1977).

#### 5.6.4 Summary

There are a large number of earlier studies available. Although these do not comply with current test guidelines, the following conclusions may be drawn with respect to genotoxicity: 1,1-Dimethyhydrazine induced gene mutations in Escherichia coli strains. In the Salmonella mutagenicity test, the substance unequivocally caused mutagenicity in the strains TA102 and TA98 and there is evidence of mutagenicity in mammalian cells in vitro. Chromosomal aberrations were detected in hamster CHO cells in vitro, indicating a clastogenic effect. DNA damage, including DNA adduct formation, could be shown with indicator tests in vitro and in vivo. In Drosophila melanogaster (SMART test), 1,1-dimethylhydrazine was found to be mutagenic and clastogenic. A chromosomal aberration test of limited validity yielded a positive result in the bone marrow of rats treated orally. Micronucleus tests with intraperitoneal administration yielded positive results in the spleen, liver and spermatids of mice; the results of micronucleus tests in bone marrow were negative. The dominant lethal tests are not included in the evaluation as they were not carried out according to current OECD test criteria.

Although none of the micronucleus tests allows a differentiation to be made between aneugenic and clastogenic effects and the in vivo chromosomal aberration test is considered to be of questionable validity, overall, clastogenicity can be assumed from the available data. The contradictory results concerning genotoxic effects in bacteria and mammals can probably be explained by the high toxicity of the substance. Furthermore, negative results in soma and germ cells in vivo can presumably be attributed to the fact that the intermediates formed from 1,1-dimethylhydrazine in the metabolism are so highly reactive that they interact rapidly locally and thus may not reach the investigated target tissue in the body. This explanation for contradictory results in genotoxicity tests has been assumed, for example, also for dimethylnitrosamine (Bruce and Heddle 1979) and suggested also for 1,2-dimethylhydrazine (Vanhauwaert et al. 2001). The accessibility of male germ cells via systemic distribution has been demonstrated.

### 5.7 Carcinogenicity

#### 5.7.1 Short-term studies

In in vitro experiments with human fibroblasts, cell transformation was observed at concentrations of 30 to 500 µM 1,1-dimethylhydrazine and during an incubation period of 6 hours (Kumari et al. 1985).

Seven to eight-week-old CDF1 mice were given 1,1-dimethylhydrazine in aqueous solution once a week for 8 weeks either by gavage (females 0.6–2.4 mg in 0.2 ml) or by intraperitoneal injection (males 0.3–1.2 mg in 0.1 ml). The doses were reduced due to toxicity in weeks 2 and 3. The total dose was 7.2 mg/mouse (females) and 3.6 mg/mouse (males). For a body weight of 28 g, this corresponded to doses of about 32 and 16 mg/kg body weight and week (4.6 and 2.3 mg/kg body weight and day, respectively). The control groups were given saline solution. Ten male and 10 female animals were used for the control groups and 30 male and 30 female animals for the treated groups. By the end of the study, 17% of the treated female animals and none of the males had died. In the control groups, mortality after oral and intraperitoneal administration of saline was 0% and 10%, respectively. Up to week 32 after exposure, the lung tumour incidence in the exposed mice was not increased with statistical significance compared with that in the respective control group (Kelly et al. 1969). Due to the short exposure period and study duration, the study cannot be included in the evaluation of carcinogenicity.

Nine-day-old ICR mice were given a single intraperitoneal injection with either a buffer solution or 20 mg 1-methylnitrosourea/kg body weight as tumour initiator. Thereafter, once a week one group received subcutaneous injections with saline and another group received injections with 20 mg 1,1-dimethylhydrazine/kg body weight. After 30 weeks, the surviving mice were examined. It was found that 1,1-dimethylhydrazine tended to increase the frequency of lung adenomas in female mice, and a tumour-promoting effect was attributed to the substance (Tamura et al. 1999).

#### 5.7.2 Long-term studies

##### 5.7.2.1 Inhalation

The data from carcinogenicity studies with 1,1-dimethylhydrazine after inhalation are listed in Table 4.

In an inhalation study, groups of 400 female mice (C57BL/6), 200 male rats (F344) and 200 male Syrian golden hamsters and 4 male and 4 female dogs (beagle) were exposed to 1,1-dimethylhydrazine contaminated with 0.12% dimethylnitrosamine. The 1,1-dimethylhydrazine concentrations used were 0, 0.05, 0.5 and 5 ml/m^3^ (0, 0.13, 1.3 and 13 mg/m^3^). The animals were exposed for 6 hours daily, on 5 days per week, for 6 months. The rodents were followed up for 18 months and the dogs for 5 years. The non-neoplastic effects are described in Section 5.2.1. Two groups of 8 female dogs, 400 female mice and 200 male hamsters served as controls. Only one control group of 200 male rats was used. In the mice exposed to 1,1-dimethylhydrazine concentrations of 0.5 and 5 ml/m^3^, there was a statistically significant increase in the number of thyroid follicular cell carcinomas. Statistically significant increases in the number of haem­angiosarcomas and Kupffer cell sarcomas were observed at concentrations of 0.05 and 5 ml/m^3^. In rats, adenomas of the pancreas and pituitary gland developed at the 1,1-dimethylhydrazine concentration of 0.05 ml/m^3^ and bronchiolar adenomas at 5 ml/m^3^ (Haun et al. 1984).

To investigate whether dimethylnitrosamine is responsible for the tumours in the studies described above, mice (C57BL/6, 200 females per group) were exposed to purified 1,1-dimethylhydrazine (< 35 µg dimethylnitrosamine/l) at concentrations of 0 or 5 ml/m^3^. The animals were exposed for 6 hours daily, on 5 days per week, for a total of 12 months with a follow-up period of 12 months. Tumours were found in the exposed animals in the lungs, liver, sinus cavities, bones, and cardiovascular and lymphatic systems. The highest tumour incidences occurred in the lungs, liver and nasal mucosa. Dimethylnitrosamine was thus not responsible for the tumours found in the other studies (Haun et al. 1984). However, the interpretation of these results is limited because in this study only one exposure concentration was chosen and only one sex of one species was exposed. Also the exposure duration was relatively short and the non-carcinogenic end points were insufficiently investigated.

**Tab.4:** Studies of the carcinogenicity of 1,1-dimethylhydrazine (inhalation)

Author:	Haun et al. 1984				
Substance:	1,1-dimethylhydrazine (0.12% DMNA as impurity)				
Species:	**rat**, F344/N, 200 ♂ per group **mouse**, C57BL/6J, 400 ♀ per group, 800 controls **hamster**, Syrian golden hamster, 200 ♂ per group, 400 controls **dog**, beagle, 4 ♂, 4 ♀ per group, 16 ♀ controls				
Administration route:	inhalation				
Concentration:	0, 0.05, 0.5, 5 ml/m^3^				
Duration:	6 months, 5 days/week, 6 hours/day, follow-up period 18 months for rodents, 5 years for dogs				
Toxicity:	non-neoplastic effects see Section 5.2.1				
**rats**		**Exposure concentration (ml/m^3^)**			
		**0**	**0.05**	**0.5**	**5**
survivors^a)^	♂	200/200	196/200	200/200	199/200
**tumours**					
bronchiolar adenomas	♂	5/189 (3%)	0/192	2/182 (1%)	10/191 (5%)**
pancreas (islet cell adenomas)	♂	0/170	3/174 (2%)**	12/169 (7%)**	6/158 (4%)**
pituitary gland (chromophobe adenomas)	♂	60/171 (35%)	76/182 (42%)**	75/169 (44%)**	90/174 (52%)**
**mice**		**Exposure concentration (ml/m^3^)**			
		**0**	**0.05**	**0.5**	**5**
survivors^a)^	♀	769/800	377/400	387/400	391/400
**tumours**					
thyroid follicular cell carcinomas	♀	2/551 (0.4%)	1/311 (0.3%)	8/278 (3%)**	5/286 (2%)**
haemangiosarcomas	♀	5/701 (0.7%)	9/374 (2%)**	3/368 (0.8%)	17/360 (5%)**
Kupffer cell sarcomas	♀	1/701 (0.1%)	4/374 (1%)**	0/368	8/360 (2%)**
No treatment-related neoplasms found in exposed **dogs** and **hamsters**					
Author:	Haun et al. 1984				
Substance:	1,1-dimethylhydrazine (< 35 μg DMNA/l as impurity)				
Species:	**mouse**, C57BL/6J, 200 ♀ per group				
Administration route:	inhalation				
Concentration:	0, 5 ml/m^3^				
Duration:	12 months, 5 days/week, 6 hours/day, follow-up period 12 months				
Toxicity:	non-neoplastic effects see Section 5.2.1				
		**Exposure concentration (ml/m^3^)**			
		**0**		**5**	
survivors^b)^	♀	about 90%		about 90%	
**tumours**					
lungs (alveolar, bronchiolar adenomas)	♀	4/187 (2.1%)		20/186 (10.7%)**	
liver (hepatocellular adenomas)	♀	4/187 (2.1%)		20/188 (10.6%)*	
lymphatic system (malignant lymphomas)	♀	64/191 (33.5%)		84/190 (44.2%)*	
		**Exposure concentration (ml/m^3^)**			
		**0**		**5**	
survivors^b)^	♀	about 90%		about 90%	
nasal mucosa papillomas	♀	0/183		5/179 (2.8%)**	
adenomatous polyps	♀	0/183		17/179 (9.5%)**	
bones (osteomas)	♀	0/183		5/179 (2.8%)*	
cardiovascular system (haemangiomas)	♀	6/191 (3.1%)		19/190 (10.0%)*	

*p ≤ 0.05

**p ≤ 0.01

DMNA: dimethylnitrosamine

a) after 6 months

b) after 12 months, no significant difference even at the end of the study (about 40% survived in both groups)

##### 5.7.2.2 Oral administration

The data from oral carcinogenicity studies with 1,1-dimethylhydrazine are listed in Table 5.

Seventy F344 rats per sex and group were given 1,1-dimethylhydrazine in concentrations of 0, 1, 50 or 100 mg/l with the drinking water (doses of 0, 0.07, 3.2 or 6.2 mg/kg body weight and day for the male rats and 0, 0.1, 4.5 or 7.9 mg/kg body weight and day for the female rats) for 24 months. Mortality in the males was 36%, 36%, 28% and 18% and in the females 32%, 24%, 28% and 10%, respectively. The number of pituitary adenomas was increased in a statistically significant manner in the high dose group of females and the sum of hepatocellular adenomas and carcinomas was increased in a statistically significant manner in the females of the middle and high dose groups. No treatment-related neoplasms were observed in the male animals (US EPA 2009). All tumour incidences are given only in percentages in the description of the results, and the study is not available in the original.

Groups of 85 and 25 female Swiss mice were given gavage doses of 0 or 5 mg 1,1-dimethylhydrazine (purity not speci­fied) on 5 days per week for 40 weeks. The 1,1-dimethylhydrazine dose was 142 mg/kg body weight and day. Lung adenomas and adenocarcinomas were found in 1/8 and 4/9 of the 40−50 and 50−60 week survivors, respectively, compared with 2/37 and 6/42 in the controls over the same time periods (Roe et al. 1967). These differences were not statistically significant. Due to the short duration of the experiment, the result cannot be used to assess the carcinogenicity of the substance.

CD-1 mice (90 animals per sex and group) were given 1,1-dimethylhydrazine (100% purity) in concentrations of 0, 40 or 80 mg/l drinking water for 24 months. The mean intake was 0, 7.34 or 13.01 mg/kg body weight and day for the male mice and 0, 11.59 or 21.77 mg/kg body weight and day for the female mice. The mortality of the animals was 70%, 76% and 98% for the males and 58%, 92% and 92% for the females. Lung and liver tumours occurred in a dose-dependent manner in both sexes. Hepatic haemangiomas and haemangiosarcomas were the most sensitive neoplastic effect. The incidences of alveolar and bronchiolar tumours likewise increased with increasing doses in the male and female animals, but not to the same extent as the neoplasms observed in the liver (US EPA 2009). The high mortality of the control animals and the high dose used make interpretation of the results of these studies difficult. Moreover, all tumour incidences are given only in percentages and the original study is not available.

In another study, CD-1 mice (90 animals per group and sex) were given 1,1-dimethylhydrazine with the drinking water. The mean intake was 0, 0.19, 0.97 or 1.9 mg/kg body weight and day for the males and 0, 0.27, 1.4 or 2.7 mg/kg body weight and day for the females. Ten mice per sex and group were studied after 6, 12, 18 and 24 months. A statistically significant increase in mortality was observed only in the males of the high dose group. Statistically significant increases in the incidences of alveolar and bronchiolar adenomas (41%) and carcinomas (14%) were found only in the females of the high dose group (US EPA 2009). The original study is not available.

Swiss mice (50 animals per sex, 110 control animals per sex) were given 0% or 0.01% 1,1-dimethylhydrazine with the drinking water (0 or 100 mg/l) continuously for 120 weeks. As the authors did not report the body weights of the animals, an average 1,1-dimethylhydrazine intake of 19 and 20 mg/kg body weight and day, respectively, was calculated based on the reference body weights of male and female mice (US EPA 2009). The survival of the treated mice was lower than that of the control animals; the reduction was statistically significant. All treated male mice died after 65 to 75 weeks and all exposed females after 55 to 65 weeks. Statistically significant increases in the number of the following tumour types were observed: angiosarcomas, the sum of lung adenomas and carcinomas, renal adenomas and benign hepatomas. Angiosarcomas were found predominantly in the liver and were described as the most sensitive neoplastic end point. The incidences of tumours of the blood vessels, lungs, kidneys and liver were 79%, 71%, 10% and 6%, respectively, for the treated male and female animals combined (Toth 1973). Limitations of this study are the unspecified purity of the substance and the use of only one dose, which was above the maximum tolerable dose.

In another study, 1,1-dimethylhydrazine was given to Syrian golden hamsters with the drinking water. Groups of 100 male and 100 female animals served as controls and 50 animals of each sex were given 0.1% (1000 mg/l) of the substance with the drinking water until death. Based on the reference values for body weights and water intake for male and female hamsters, an average 1,1-dimethylhydrazine intake of 134 and 131 mg/kg body weight and day, respectively, was calculated (US EPA 2009). The survival of the treated hamsters was lower than that of the controls. All treated male hamsters had died by weeks 84 to 94 and all treated females by weeks 74 to 84 (US EPA 2009). Caecum adenomas and adenocarcinomas were observed in 14 and 2 of the 50 treated male and female animals, respectively. Angiomas and angiosarcomas, mainly in the liver, were observed in 15 and 10 of the 50 treated animals of both sexes, respectively. An increased number of adrenal cortical adenomas were found in the treated females (in 4 of 50 animals) (Toth 1977). Limitations of this study are the unspecified purity of the substance and the use of only one dose, which was above the maximum tolerable dose.

##### 5.7.2.3 Subcutaneous administration

European hamsters (MHH:EPH) were given 1,1-dimethylhydrazine via subcutaneous injection once a week in doses of 3.73 mg/kg body weight and day (males) and 3.25 mg/kg body weight and day (females) until death. A total of 15 male and 15 female animals were treated. The control group consisted of 8 animals of each sex that received only saline. The substance-related formation of malignant peripheral nerve sheath tumours (neurofibrosarcomas, Schwann cell melanomas) was diagnosed (Table 5; Ernst et al. 1987).

**Tab.5:** Studies of the carcinogenicity of 1,1-dimethylhydrazine (oral and subcutaneous uptake)

Author:	US EPA 2009						
Substance:	1,1-dimethylhydrazine						
Species:	**rat**, F344, groups of 70 ♂ and 70 ♀						
Administration route:	drinking water						
Concentration:	0, 1, 50, 100 mg/l (♂/♀: 0.07/0.1, 3.2/4.5, 6.2/7.9 mg/kg body weight and day)						
Duration:	2 years						
Toxicity:	none						
		**Dose (mg/kg body weight and day) ♂/♀**					
		**0**	**0.07/0.1**		**3.2/4.5**		**6.2/7.9**
survivors	♂	64%	64%		72%		82%
	♀	68%	76%		72%		90%
**tumours**							
pituitary adenomas	♀	32%	no data		no data		56%**
hepatocellular adenomas	♀	0%	2%		4%		2%
hepatocellular carcinomas	♀	0%	0%		6%		8%
liver tumours (sum)	♀	0%	no data		10%**		10%**
Author:	US EPA 2009						
Substance:	1,1-dimethylhydrazine						
Species:	**mouse**, CD-1, groups of 90 ♂ and 90 ♀						
Administration route:	drinking water						
Concentration:	0, 40, 80 mg/l (♂/♀: 7.34/11.59, 13.71/21.77 mg/kg body weight and day)						
Duration:	2 years						
Toxicity:	mortality ↑						
		**Dose (mg/kg body weight and day) ♂/♀**					
		**0**		**7.34/11.59**		**13.71/21.77**	
survivors	♂	27/90		22/90		2/90	
	♀	38/90		7/90		7/90	
**tumours**							
hepatic haemangiomas and haemangiosarcomas	♂	9%		67%		81%	
	♀	4%		26%		82%	
alveolar and bronchiolar tumours	♂	18%		45%		55%	
	♀	14%		50%		48%	
no data for significance given in US EPA (2009)							
Author:	US EPA 2009						
Substance:	1,1-dimethylhydrazine						
Species:	**mouse**, CD-1, groups of 90 ♂, 90 ♀						
Administration route:	drinking water						
Concentration:	0, 1, 5, 10 mg/l (♂) and 0, 1, 5, 20 mg/l (♀) (♂/♀: 0.19/0.27, 0.97/1.4, 1.9/2.7 mg/kg body weight and day)						
Duration:	2 years						
Toxicity:	mortality ↑						
		**Dose (mg/kg body weight and day) ♂/♀**					
		**0**	**0.19/0.27**		**0.97/1.4**		**1.9/2.7**
survivors	♂	52%	46%		48%		32%
	♀	40%	50%		46%		24%
**tumours**							
alveolar and bronchiolar adenomas	♀	5/49 (10.2%)	no data		no data		20/49 (40.8%)**
alveolar and bronchiolar carcinomas	♀	1/49 (2%)	no data		no data		7/49 (14.3%)**
Author:	Toth 1973						
Substance:	1,1-dimethylhydrazine						
Species:	**mouse**, Swiss Albino, groups of 50 ♂ and 50 ♀; 100 ♂ and 100 ♀ as controls						
Administration route:	drinking water						
Concentration:	0, 100 mg/l (♂/♀: 19/20 mg/kg body weight and day)						
Duration:	120 weeks						
Toxicity:	mortality ↑						
		**Dose (mg/kg body weight and day) ♂/♀**					
		**0**			**19/20**		
survivors	♂	0 after weeks 115–125			0 after weeks 65–75		
	♀	0 after weeks 105–115			0 after weeks 55–65		
**tumours**							
angiosarcomas	♂	2/110 (1.8%)			42/50 (84%)*		
	♀	4/110 (3.6%)			37/50 (74%)*		
lung adenomas and carcinomas	♂	11/110 (10%)			39/50 (78%)*		
	♀	14/110 (12.7%)			32/50 (64%)*		
renal adenomas	♂	0/110			1/50 (2%)		
	♀	0/110			9/50 (18%)*		
benign hepatomas	♂	0/110			6/50 (12%)*		
	♀	0/110			0/50		
Author:	Toth 1977						
Substance:	1,1-dimethylhydrazine						
Species:	**Syrian golden hamster**, groups of 50 ♂ and 50 ♀; 100 ♂ and 100 ♀ as controls						
Administration route:	drinking water						
Concentration:	0, 1000 mg/l (♂/♀: 134/131 mg/kg body weight and day)						
Duration:	lifetime						
Toxicity:	mortality ↑						
		**Dose (mg/kg body weight and day) ♂/♀**					
		**0**			**134/131**		
survivors	♂	0 after weeks 104–114			0 after weeks 84–91		
	♀	0 after weeks 84–94			0 after weeks 74–84		
**tumours**							
angiomas and angiosarcomas	♂	0/100			15/50 (30%)*		
	♀	1/100 (1%)			10/50 (20%)*		
caecum adenomas and adenocarcinomas	♂	0/100			14/50 (28%)*		
	♀	0/100			2/50 (4%)		
adrenal adenomas	♂	4/100 (4%)			0/50		
	♀	1/100 (1%)			4/50 (8%)*		
Author:	Ernst et al. 1987						
Substance:	1,1-dimethylhydrazine						
Species:	**European hamster**, MHH:EPH, groups of 15 ♂ and 15 ♀; 8 ♂ and 8 ♀ as controls						
Administration route:	weekly subcutaneous injection						
Dose:	0, ♂/♀: 3.73/3.25 mg/kg body weight						
Duration:	lifetime						
Toxicity:	mortality ↑						
		**Dose (mg/kg body weight and week) ♂/♀**					
		**0**			**3.73/3.25**		
survivors^a)^	♂	105 weeks			72 weeks		
	♀	101 weeks			85 weeks		
**tumours**							
animals with a tumour	♂	3/8			10/14		
	♀	5/8			9/15		
malignant nerve tumours (%)	♂	0			43		
	♀	0			40		
other tumours^b)^	♂	3			6		
	♀	5			14		

*p < 0.05, Fisher’s exact test (US EPA 2009)

**statistically significant, p value not reported

a) average survival

b) exposed male animals: hepatocellular adenomas (1), renal adenomas (1), adrenal adenomas (1), phaeochromocytomas (1), leukaemia (1), prostate adenocarcinomas (1); exposed female animals: granulosa cell tumours (5), dermal melanomas (2), hepatocellular carcinomas (2), gastric adenocarcinomas (2), renal adenocarcinomas (2), salivary gland adenocarcinomas (1), reticular cell sarcomas in spleen (1); males in control group: forestomach papillomas (1), phaeochromocytomas (1), malignant lymphomas (1); females in control group: granulosa cell tumours (1), adrenal adenomas (1), cholangioadenomas (1), malignant lymphomas (1)

#### 5.7.3 Summary

Although all available studies can be used for the evaluation only to a limited extent, overall, the data show that 1,1-dimethylhydrazine is carcinogenic. In inhalation studies in rats, islet cell adenomas occurred, and in mice thyroid carcinomas, haemangiosarcomas and Kupffer cell sarcomas. In a study with pure 1,1-dimethylhydrazine, there was an increased incidence of lung and liver adenomas, lymphomas, nasal mucosal adenomas, osteomas and haemangiomas.

Ingestion of the substance resulted in pituitary adenomas and hepatocellular adenomas and carcinomas in rats. In mice, haemangiomas, haemangiosarcomas, renal adenomas and lung tumours were observed. In hamsters, angiomas and angiosarcomas, caecum tumours, adrenal adenomas and, when administered subcutaneously, malignant nerve tumours developed.

### 5.8 Other effects

Female and male BALB/c mice given intraperitoneal injections of 1,1-dimethylhydrazine in doses of 10 or 50 mg/kg body weight 3 times per week for 14 weeks exhibited an increase in the production of antibodies to sheep erythrocytes. At 50, 100 and 150 mg/kg body weight, reduced T-cell induction by concanavalin A was observed (Tarr et al. 1982). In mice, treatment with 1,1-dimethylhydrazine resulted in increased one-way mixed lymphocyte response and decreased microbicidal activity, chemotaxis and the production of prostaglandin E2 in vitro in macrophages (Tarr 1989; Tarr et al. 1988).

## 6 Manifesto (MAK value/classification)

The critical effect is the carcinogenic effect due to genotoxicity.

**Carcinogenicity. **In employees of a rocket engine test facility who were exposed to 1,1-dimethylhydrazine, among other substances, it cannot be ruled out that exposure to 1,1-dimethylhydrazine made a contribution to an increased cancer risk. These data support the assumption that the substance causes carcinogenic effects in humans; however, the existing data are not sufficiently reliable to be used as valid evidence. In inhalation studies, benign and malignant tumours were found in numerous organs in mice and rats. Tumours occurred likewise after oral administration, also in hamsters. Inhalation or oral administration of pure 1,1-dimethylhydrazine caused marked tumour development in mice (nasal mucosa, lungs, liver, lymphatic system, bones and blood vessels); the dimethylnitrosamine present in commercial 1,1-dimethylhydrazine as an impurity is therefore not responsible for the carcinogenic effect. The substance is mutagenic and clastogenic. The classification of 1,1-dimethylhydrazine in Carcinogen Category 2 has therefore been retained.

**MAK value. **Cysts in the endometrium and hyaline degeneration of the gallbladder of mice were observed after exposure to the lowest concentration teste^d^ of 0.05 ml/m^3^ for 6 months (Haun et al. 1984). As the histopathological examination was not carried out until 18 months after the end of exposure, a NOAEC cannot be derived. A genotoxic mode of action is of prime importance for carcinogenicity. The data from the oral carcinogenicity studies described in US EPA (2009) cannot be used to establish an exposure–risk relationship or indicate a risk because the original data are not available. A MAK value cannot be derived and the substance is thus not assigned to a peak limitation category.

**Prenatal toxicity. **In developmental toxicity studies, an increased number of resorptions, a reduction in foetal weights and occasional skeletal and visceral malformations of the foetuses were observed after intraperitoneal administration in pregnant rats. Due to the intraperitoneal injection, a direct effect of the substance on the foetus cannot be ruled out. Therefore, the study is not included in the evaluation of the substance. As a MAK value cannot be derived, assignment to a pregnancy risk group is not applicable.

**Germ cell mutagenicity. **1,1-Dimethylhydrazine is mutagenic in bacteria and mammalian cells. Mutagenicity tests in vivo are not available. Based on in vitro and in vivo studies, an alkylating effect of 1,1-dimethylhydrazine on DNA can be assumed. Although none of the available micronucleus tests allows a differentiation to be made between aneugenic and clastogenic effects and the in vivo chromosomal aberration test is considered to be of questionable validity, a clastogenic effect in somatic cells is to be assumed in the light of all the available data. Because of the intraperitoneal administration route, the positive result in the micronucleus test with mouse spermatids is not proof of germ cell mutagenicity in vivo. The accessibility of germ cells could be shown, however, in a study with oral administration. On the basis of all the available data, 1,1-dimethylhydrazine has been classified in Category 3 A for germ cell mutagens.

**Absorption through the skin. **Valid studies of the absorption of 1,1-dimethylhydrazine through the skin are not available. The dermal LD_50_ values are in the range of 1000 mg/kg body weight. Since 1,1-dimethylhydrazine is a carcinogen with genotoxic properties and dermal absorption can be assumed to contribute to the genotoxic risk, designation with an “H” (for substances which can be absorbed through the skin in toxicologically relevant amounts) has been retained.

**Sensitization. **1,1-Dimethylhydrazine has been designated with “S” and “Sh” (for substances which cause sensitization of the skin) since 1973. However, there are no data available for sensitizing effects on the skin or the respiratory tract. According to the criteria for the evaluation of sensitizing substances (see List of MAK and BAT Values, Section IV (DFG 2021)), a close structural relationship with similar substances that have been classified as sensitizing substances is not sufficient in itself to assume that the substance is likely to have a sensitizing effect if additional positive findings are not available. However, in the case of the methylhydrazines, it seems plausible that they would cause contact sensitization because of the close structural similarity to hydrazine, which is known to be a pronounced contact allergen and has been designated with “Sh”. 1,1-Dimethylhydrazine was assigned the designation “Sh” in 1973 as a precautionary measure. This designation has been retained because there are no data available that demonstrate that 1,1-dimethylhydrazine is not a contact allergen.
